# Hypothesis: Astrocyte Foot Processes Detachment from the Neurovascular Unit in Female Diabetic Mice May Impair Modulation of Information Processing—Six Degrees of Separation

**DOI:** 10.3390/brainsci9040083

**Published:** 2019-04-14

**Authors:** Melvin R. Hayden

**Affiliations:** 1 Diabetes and Cardiovascular Center, University of Missouri School of Medicine, Columbia, MO 65212, USA; mrh29pete@gmail.com; 2 Division of Endocrinology and Metabolism, Department of Medicine, University of Missouri, Columbia, MO 65212, USA

**Keywords:** astrocyte, basement membrane, astrocyte foot processes, endothelial cells, pericyte’s, neurovascular unit, neurovascular coupling, neuron

## Abstract

Astrocytes via their foot processes (ACfp) are specialized connecting cells, and they structurally connect the neurovascular unit (NVU) mural cells to neurons. Astrocytes provide homeostatic mechanisms for structural connections and provide communication between the NVU and regional neurons for functional hyperemia in regions of increased neuronal activity (neurovascular coupling). Previously, our group has demonstrated a detachment, separation, and retraction of ACfp in diabetic *db*/*db* females (DBC). It was hypothesized that a loss of adherent ACfp/NVU could result in the known impaired cognition in DBC. Additionally hypothesized was that empagliflozin treatment could protect DBC ACfp/NVU remodeling. This study demonstrates a significant loss of ACfp/NVU numbers in DBC and a protection of this loss by empagliflozin treatment (DBE). The number of intact ACfp/NVU was 6.45 ± 1.1 in control heterozygous (CKC) vs. 1.88 ± 0.72 in DBC (*p* < 0.05) and 5.86 ± 0.88 in DBE vs. DBC (*p* < 0.05) by visually hand-counting the capillary NVUs (22 in CKC, 25 in DBC, and 22 in DBE). These findings suggest that empagliflozin provides neuroprotection via the prevention of ACfp separation in DBE as compared to diabetic DBC. Furthermore, a loss of ACfp/NVU numbers in DBC may correspond with a negative modulation of informational processing, and the protection of ACfp/NVU numbers could provide a protective modulation in DBE models.

## 1. Introduction

Type 2 diabetes mellitus (T2DM) is an age-related disease strongly associated with obesity (especially visceral obesity), insulin resistance, and metabolic syndrome [1,2,3,4,5,6]. Furthermore, T2DM is known to be a risk factor for the development of age-related late-onset Alzheimer’s disease-dementia (LOAD) [1,5,6]. Similar to T2DM, the sporadic age-related LOAD is also related to the same above risk factors for T2DM. Both T2DM and LOAD are now considered to be global epidemic phenomena. The alarming parallel of these two pandemics will place a tremendous financial burden on our health care system workers and providers, as well as, family care providers in the coming decades as the global post-World War II baby boom generation expands exponentially over the next two decades [1,2,3,4].

The goal of this short communication is to focus on the protoplasmic layer III cortical astrocytes and the number of astrocyte end-feet foot processes (ACfp) per capillary neurovascular unit(s) (NVU) (ACfp/NVU) with aberrant ultrastructural phenotypic remodeling and to discuss the ACfp/NVU losses and protection as well as how these changes may affect the known impaired cognition and possible impaired modulation of informational processing as a result of this loss and the protection of this modulation with the antidiabetic drug empagliflozin.

The specific aim is to count the number of ACfp/NVU in each of the control nondiabetic (CKC), the *db*/*db* diabetic (DBC), and the DBC treated for 10 weeks with empagliflozin (DBE) models. The transmission electron microscope (TEM) aberrant remodeling changes may be characterized by ACfp detachment, separation, and retraction from the mural capillary neurovascular unit (NVU) endothelial cell(s) (EC) and from the vasoactive (contractile/relaxation) properties of pericyte(s) (Pc) and their shared outer basement membrane(s) (BM) [1,4]. These TEM aberrant remodeling changes are especially important, since it is now known that the *db*/*db* diabetic model has an impaired cognition [1,5,6] and that empagliflozin, a sodium glucose transporter inhibitor (SGLT2i), is capable of preventing this cognitive dysfunction [5].

## 2. Methods

Animal studies, tissue location, sample collection, preparation for TEM, and regions of interest have been described for each of the animal models discussed [1,2,3,4].

### Image Acquisition and Counting of Astrocyte Foot Processes in Each Model

The images for this study were derived from the same cohort of animals previously presented [1,2,3,4]. The monogenic *db*/*db* (Lepr^db^) female diabetic *db*/*db* (BKS.CgDock7^m^ +/+ Lepr^db^/J) (DBC) model was utilized, and comparisons were made to the control CKC and treated DBE models. Briefly, these cohorts were examined specifically to count the total number of tightly adherent ACfp in capillary NVUs (22–25) in each model of the heterozygous nondiabetic control (CKC) (22), homozygous diabetic *db*/*db* (DBC) (25), and the DBC (22) treated with empagliflozin (DBE) from 10 weeks of age to 20 weeks of age at sacrifice (*n* = 3) for each model with 7–8 capillary NVUs/model. The tightly adherent ACfp were carefully and visually identified and hand-counted by the author at various magnifications (varying from ×2500 to ×4000 with scale bars between 0.5–1.0 micrometers) in order to include the entire capillary NVU (blinded) and recorded for each model; then unblinded and compared with the Student *t*-test, with *p*-values less than 0.05 being significant. The results are reported as the mean ± SE with error bars.

## 3. Results

**Figure 1:**
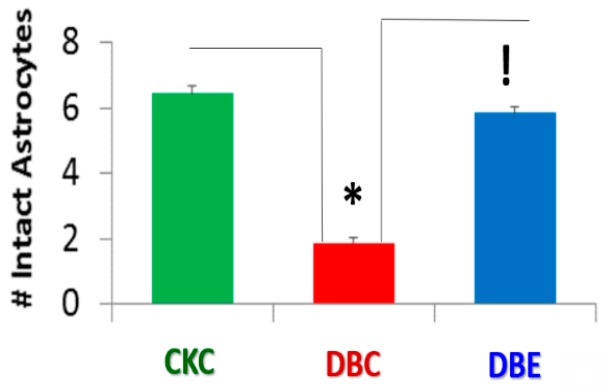
The diabetic *db/db* models have a significant decrease in the number of intact and attached astrocyte foot processes (ACfp) at the outer basement membrane of the capillary neurovascular unit (NVU): This image represents the mean number (#) of astrocytes (ACfp)/NVU that are firmly attached and abut to the outer endothelial and pericyte outer basement membranes of the capillary neurovascular unit (NVU), numbering 22 in CKC (control heterozygous: green), 25 in DBC (diabetic *db/db*: red), and 22 in *db*/*db* empagliflozin-treated (DBE: blue) for a total of 69 capillary NVUs counted. The mean number of ACfp/NVU was 6.4 ± 1.1 in CKC, 1.88 ± 0.72 in diabetic DBC, and 5.86 ± 0.88 in diabetic *db*/*db* treated with the SGLT2 inhibitor (empagliflozin) DBE. * CKC compared to DBC (*p*-value < 0.05); ! DBC compared to DBE (*p*-value < 0.05).

In previous publications, we had only shared our marked observational findings regarding the detachment, separation, and retraction of ACfp and felt it was necessary to focus on the possibilities of performing a semiquantitative study regarding the number of ACfp tightly adhered in control CKC and detached and separated in DBC and to determine semiquantitatively the number of tightly adhered ACfp/NVU in empagliflozin-treated DBC. The current study demonstrated a significant loss in the number of intact ACfp/NVU in untreated diabetic DBC as compared to the control CKD (6.45 ± 1.1 ACfp/NVU in CKC as compared to 1.88 ± 0.72 ACfp/NVU in DBC) (*p* < 0.05), and the protection with an empagliflozin treatment of DBC resulted in 5.86 ± 0.88 ACfp/NVU in DBE (*p* < 0.05) (Figure 1).

## 4. Discussion

Previous publications [1,2,3,4,5,6] and the preceding introduction and background information prompted the notion to view the brain as an end organ of the monogenic *db/db* (*Lepr^db^*) female diabetic *db*/*db* (BKS.Cg*Dock7^m^* +/+ *Lepr^db^*/J) (DBC) model in respect to the astrocytes (AC) and the number of ACfp/NVU. The female DBC [1,2,3,4] and its male counterpart [5,6] are well-known, extensively studied and accepted models of T2DM, obesity, and insulin resistance [1,2,3,4,5,6]. For example, when using the search terms “diabetes”, “diabetic *db*/*db* mouse model”, or “*db*/*db*”—“*db*/*db* brain” on the US National Library of Medicine National Institutes of Health—US National Library of Medicine PubMed data base search engine, there were 878, 3594, and 380 entries respectively.

Additionally, the NVU is readily visualized by TEM ultrastructural studies including the ACfp and the NVU as well as the other neuroglia cells of the NVU, neuropile, and neurons. Previously, our group published a 3-part trilogy series of References [1,2,3] examining the specific role of the NVU, neuroglia, and neurons with a focus on the astrocyte in part I, on the microglia and mitochondria in part II, and on the oligodendrocyte and neuronal axon myelin in part III [1,2,3]. In our newly published paper in the Neuroglia Section of Brain Sciences [4], we were able to share our findings regarding the treatment and the protection provided to the NVU, neuroglia, and neuronal axons by the glucose-lowering effects of the clinically available antidiabetic glucose-lowering medication empagliflozin (an SGLT2 inhibitor).

**Figure 2:**
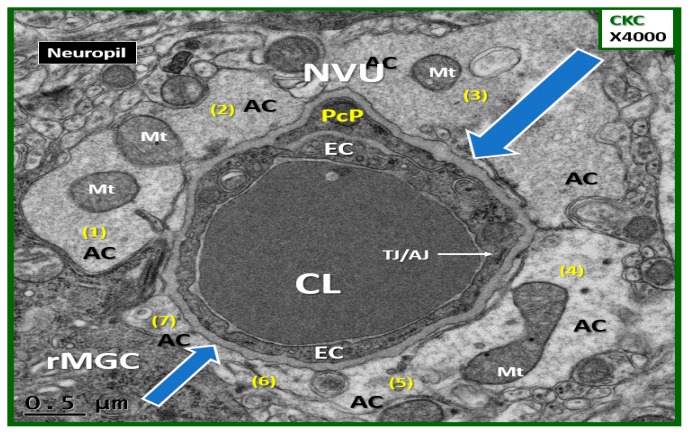
A representative image of the control CKC nondiabetic model with intact adherent astrocytes: This image depicts the astrocyte end-feet foot processes (ACfp) numbering seven (1–7 in yellow) that are tightly abutted and adherent to the basement membrane (BM) (open blue arrows) of the NVU mural endothelial cell(s) (EC) and pericyte end-feet foot processes (Pc). Note the one tight and adherent junction (TJ/AJ) in this cross-sectional image (closed arrow). Magnification ×4000; scale bar = 0.5 µm. AC = astrocytes; CL = capillary lumen; EC = endothelial cell; Pc = pericyte; rMGC = ramified microglia cell; NVU = neurovascular unit TJ/AJ = tight junction/adherent junction. Available via license CC BY 4.0.

**Figure 3:**
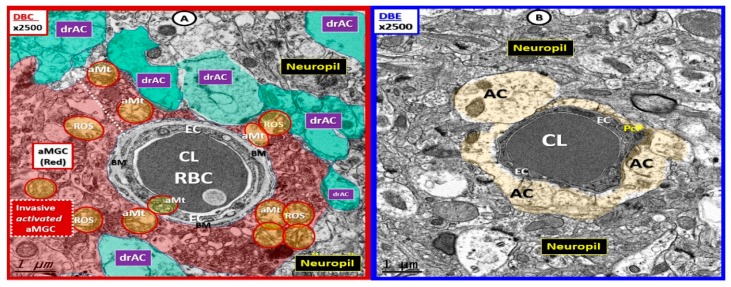
The basement-membrane-thickening, activated microglia invasion and detached, separated astrocyte foot processes are protected with empagliflozin. (**A**) The invasion of the activated microglia cell (aMGC) to totally engulf the neurovascular unit (NVU) (uncolored) with a detachment-separation of the AC end foot processes (drAC = detached, retracted, and separated astrocyte; pseudo-colored green). Also note the aberrant mitochondria (aMt) (pseudo-colored yellow with red outline) in DBC, which may be responsible for Mt-derived reactive oxygen species (ROS) production and leakage. (**B**) In DBE, the empagliflozin protects the NVU from aberrant remodeling in the DBC as seen in Figure 3A. Note the intact AC end feet (pseudo-colored golden) and that the mitochondria in DBE are electron dense and not aberrant as in the DBC. Magnification ×2500; scale bar = 1 µm. aMGC = activated microglia cell; aMt = aberrant mitochondria; drAC = detached retracted AC EC = endothelial cell; CL = NVU capillary lumen; Pc = pericyte foot process; RBC = red blood cell; ROS = reactive oxygen species. Available via license CC BY 4.0.

**Figure 4:**
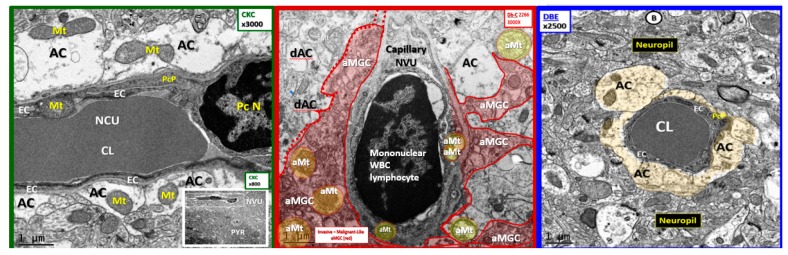
A comparison of the control, diabetic *db*/*db*, and empagliflozin-treated models: The control CKC (far left) depicts multiple tightly adherent intact astrocyte end-feet foot processes (ACfp) to the basement membrane (BM) of the capillary lumen (CL) of the neurovascular unit (NVU). Also note that the AC tightly adheres not only to the basement membranes of endothelial cells (EC) but also to the BM of the pericyte process (PcP) of the protective pericyte nucleus (PC N). Also note the very heavily elongated, electron-dense tight and adherence junctions at the endothelial cell (EC) paracellular cell–cell junctions where the ECs overlaps. The insert (lower right) demonstrates the proximity of the NVU to the regional pyramidal neuron (PYR). The middle panel demonstrates the near complete loss of ACs leaving only one intact AC that is partially detached, with the remainder ACs being detached separated and retracted (dAC). Note the activated microglial cell (aMGC pseudo-colored red) separating the ACs from the basement membrane of the NVU. The far-right panel illustrates that the treatment with empagliflozin protects the ACs from detachment, separation, and retraction and that the capillary NVU is completely encircled by a corona of six intact and tightly adherent ACs (pseudo-colored golden). The magnifications at the top of each panel, and the scale bars are at the bottom. Available via license CC BY 4.0.

Since our study regarding the effects of empagliflozin were recently published [4], the decision was made to go back and reexamine 22–25 different capillary NVUs in each of our models, which include the heterozygous nonobese, non-insulin resistant, and nondiabetic control model (CKC) (Figure 2); the *db*/*db* homozygous obese, insulin resistant, and T2DM diabetic model (DBC) (Figure 3); and the DBC treated with empagliflozin (DBE) with an *n* = 3 in each model (Figure 2, Figure 3 and Figure 4). In previous publications, it was observed that there were marked changes in the female diabetic *db*/*db* astrocytes in that they were detached and retracted from the neurovascular unit [1,4]. However, the overall importance and implications of this finding were not properly noted or addressed until the question was posed by the call for papers regarding “How can astrocytes specifically modulate information processing?”

Note that most of the TEM figures, which follow, have been previously published [1,2,3,4] either in their original or slightly altered format with either different magnifications, labeling, or arrangements in order to focus on ACfp adherence or separation and to be made available via license CC BY 4.0.

The empagliflozin treatment was observed to markedly and now semiquantitatively protect the detachment, separation, and retraction of the ACfp from the basement membrane of the two mural cells responsible for the vasodilation of the capillary NVU. This protection by empagliflozin may result in an improved cerebral blood flow due to structural and functional neurovascular coupling when regional neurons become active by various stimuli (Figure 2, Figure 3 and Figure 4).

### 4.1. Astrocytes and Astrocyte Foot Processes May Be Essential to Modulate Informational Processing in the Brain

The neuroglial ACs and their ACfp are essential for the molecular, cellular network (especially the tripartite synapse and plasticity), systemic, organ CNS-brain, and metabolic homeostasis [7]. The ACs not only provide for the requisite defense and homeostasis mechanisms but also serve as the bidirectional signaling connecting cells between regional neurons and vasoactive mural cells of the NVU, which allow the capillary NVU to provide the necessary protective neurovascular coupling function associated with a matched cerebral blood flow [7,8]. Interestingly, human astrocytes are approximately 15 times larger, have an extended outreach to neuronal synapses (approximately 2 million in humans verses 20–120 in rodents), and are thought to be more complex as compared to rodent AC [8].

**Figure 5:**
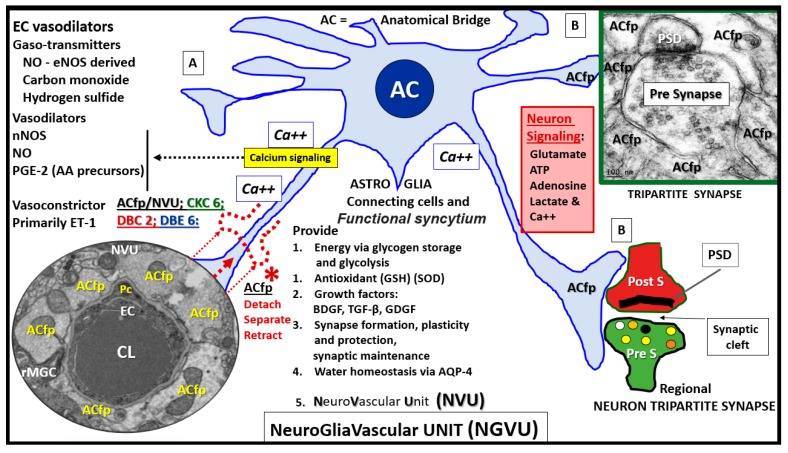
The astrocyte connectivity between the capillary and neurons to form the neurovascular unit: This image illustrates the combination of cartoon and transmission electron micrographs (TEM). (**A** and **B**) The importance of astrocyte (AC) connections via their end-feet foot processes (ACfp): The connection between the cerebral capillary neurovascular unit (NVU-magnification ×4000) and astrocyte (AC-ACfp) reside at each end of the AC colored light blue. Note that Figure 5A is the capillary NVU to the left and that the tripartite neuronal synapse on the right is Figure 5B. Note the detachment separation and retraction of the ACfp (red dashed lines) in Figure 5A. Note the red and green colorized pre- and post-synapse and the synaptic cleft (20 nm) and its relation to the connecting AC end-feet with the adjacent TEM image of a tripartite synapse, and note how the blue-colored AC end-feet are connected and tightly adherent in the control CKC models. Note the important role that the ACfp have in being tightly abutted to the asymmetrical synapse of the neuronal pre- and postsynaptic neurons of the empagliflozin-treated model in the TEM image (Figure 5B); magnification ×20,000; scale bar = 100 nm. We did not observe any loss of these connections with the TEM images to the neuronal synapse, but even with the higher magnification, it was difficult to envision at this point, and future attempts will be entertained with FIB/SEM technology. The concept to create this combination cartoon and TEM image was inspired by Verkhratsky and Nedergaard [7]. The TEM image in Figure 5B is novel, while the TEM image in Figure 5A was available via license CC BY 4.0.

The autoregulation of regional cerebral blood flow (CBF) is one of the brain’s very unique and highly specialized structural and functional mechanisms in addition to the very rigorous control of what it allows to pass from the systemic circulation to the interstitial space of the brain via the illusive EC luminal surface endothelial glycocalyx layer, the basolateral paracellular tight and adherent junction(s) (TJ/AJ) or blood–brain barrier (BBB), the pericyte (Pc), the abluminal basement membrane (BM), and the connecting bidirectional communicating ACfp to the regional neurons [1,2,3,4] (Figure 5).

### 4.2. Hypothesis: Astrocyte Foot Process Loss from Six to Two per Neurovascular Unit in Diabetic db/db Models could Result in Impaired Modulation of Neuronal Information Processing and Impaired Cognition

**Figure 6:**
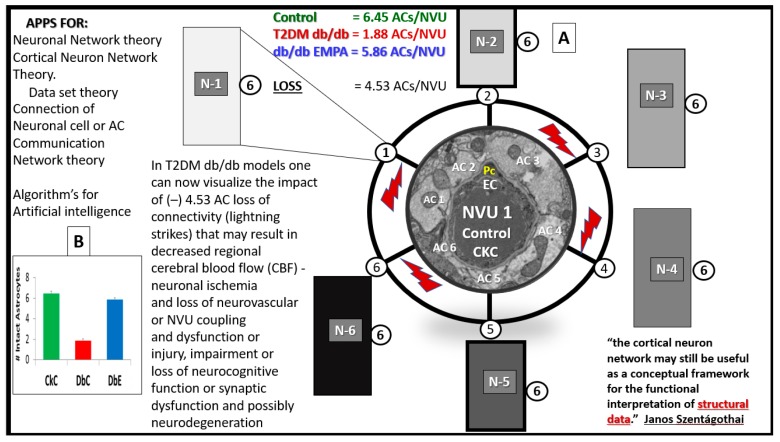
The possible mechanisms involved regarding the six degrees of separation between regional neurons and the loss of astrocyte foot processes hypothesis in diabetes: Having quantified the loss of four ACfp/NVU in the diabetic DBC as compared to CKC and DBE, one can now better envision the potential impact on regional neuron dysfunction and how that might affect extremely large numbers of connecting interneurons. The NVU is placed centrally and illustrates 6 ACfp that connect to possibly 6 connection points of regional neurons that, in turn, may connect to 6 other interneurons (**A**). This figure may allow one to better envision the potential devastating effects of just how vast the connections are within the mammalian brain and especially in humans with such a vast expansion of the neocortical synapses (approx. 0.15 quadrillion) (Figure 6A). The loss of four ACfp/NVU in the DBC is represented by red lightning strikes, and the bar graph demonstrates the loss of ACfp/NVU in the DBC (**B**). This hypothetical figure also adds to the currently well-accepted concept that a typical neuron receives on the order of 10,000 synaptic contacts. ACfp = astrocyte foot processes; APPS = applications; T2DM = type 2 diabetes mellitus; N = neuron; NVU = neurovascular unit. Available via license CC BY 4.0.

Because cells do not exist in decimal points but only in complete cells, the rounded mean number of six ACfp/NVU in CKC and DBE immediately brought to mind the highly discussed and debated “6 degrees of separation” theory that was initially entertained by Janos Szentagothai (1912–1994); the noted Hungarian neuroanatomist, electron microscopist, and neuroendocrinologist contributed to the “six degrees of separation” theory and proposed that each neuron was able to contact any other neuron with no more than six interneuronal connections. Szentagothai also promoted the cerebral cortical neuronal network via the “module-concept” and shared the following: “The cortical neuron network may still be useful as a conceptual framework for the functional interpretation of structural data.” [9]. In retrospect, this noted Hungarian was able to begin the thought process of today’s neuronal network theory and how this may possibly apply to artificial intelligence. Therefore, now it becomes plausible to also consider the “6 degrees of separation” theory-hypothesis in ACfp/NVU connections, since the mean rounded number of ACfp/NVU were six in CKC and six ACs/NVU in DBE but only 2 AC/NVU in the diabetic DBC. Furthermore, the loss of four ACfp/NVU in the DBC could have a huge negative impact on neurovascular coupling and CBF to regional neuronal activity with resulting hypoperfusion, hypometabolism, and possibly impaired cognition and neuronal dysfunction—neurodegeneration (Figure 6).

Some have estimated by stereological techniques that there may be as many as 20 billion neocortical neurons and up to 0.15 quadrillions synapses within just the neocortex [10]. It is difficult for most to fathom such large numbers; however, most of us can compare these huge numbers to the stars we see in our universe and can compare to our own Milky way galaxy of our universe, and this may be just within the neocortex. With this perspective in mind, one can begin to imagine a decrease from six ACfp/NVU in CKC to 2 ACfp/NVU in the DBC to be of important significance. As we begin to comprehend such large numbers with artificial intelligence, even these small numbers (from six to two with a loss of four ACfp/NVU) could become vitally important. Additionally, it may be even more important as to where these regions of detachment, separation, and retraction of AC end-feet are occurring. The current findings were noted in the cortical grey matter in the more frontal neocortical regions in layer III. Even when one is dealing with even smaller numbers, they may become phenomenological when the total numbers are so big. Additionally, this hypothesis with the above potential mechanisms may become more important over time, especially since we now understand that the number of ACfp can be protected in the diabetic *db*/*db* with antidiabetic medication empagliflozin. While Figure 6 proposes only a hypothesis, it should be realized that this does not mean that each cell makes connections with only the fixed six other neurons, as the numbers could be either lower or higher, especially in different regions of the brain.

### 4.3. Limitations

The following limitations apply to this current study and are similar to previous publications [1,2,3,4]. First, we fixed the tissue by an immediate immersion rather than by perfusion fixation in order to be better prepared to study the human biopsy and postmortem tissue samples. Second, our study was limited to the cortical grey matter primarily in layer III and may not apply to other regions of the brain. Third, this study was designed to directly interrogate capillary NVUs and the ACfp/NVU and only their immediate surroundings in order to count the intact, tightly adherent, detached, separated, and retracted ACfp. However, we have studied more extensively and reported on the surrounding ultrastructure of the immediate cellular and extracellular surrounding structures [1,2,3,4]. Fourth, female models were utilized in contrast to most other studies, which utilize male models; however, the female model findings seem to provide reproducible findings similar to the males [1,2,3,4]. Fifth, the counting of ACfp in models had been acquired previously by an observer, and even though blinded as to what model was in the counting process, the observer had previously seen these images and there is the slightest possibility that some bias could have entered into the counting. However, ACfp are either attached or they are separated, since those partially attached or detached were not counted. Finally, this study was limited to only ultrastructural findings, and these cohort of animals were not supported by functional studies such as functional hyperemia, immunohistochemistry, light microscopy, or protein blots. When appropriate, references were made to the *db*/*db* model from other *db*/*db* experiments that utilized the same monogenic *db*/*db* model of obesity and insulin resistance that spontaneously develops T2DM. Importantly, it is not known whether each ACpf of the NVU signals a different neuron. Regardless, the loss of adherent ACfp would impair cerebral blood flow and neurovascular coupling.

**Figure 7:**
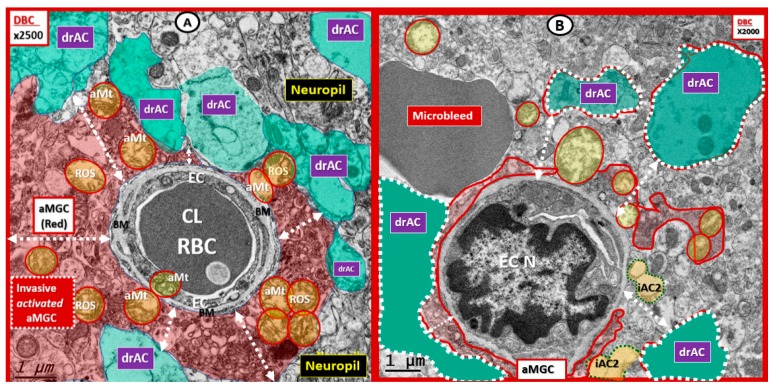
The separation, detachment, and retraction of astrocyte end-feet foot processes (ACfp) in female diabetic DBC with a loss in the connectivity to regional neurons: (**A** and **B**) Representative images of the neurovascular units (NVU) with ACfp–drAC (detached, retracted, and separated) (pseudo-colored cyan). Both images depict a basement-membrane (BM) thickening with aberrant mitochondria (aMt) pseudo-colored yellow with encircling red lines. Incidentally, aMt may be a source of reactive oxygen species (ROS). Note the invading activated MGCs (aMGC) (pseudo-colored red). In Figure 7A, the activated microglia (aMGC) has totally encompassed the NVU, while in Figure 7B, it has not quite completely undergone total invasion. Note the white dashed double arrows in each image denoting these separations of the drACs (ranging from 0.2 to 2 micrometers) from the NVU BMs. These separations of ACfp–drACs could definitely interrupt and result in a loss of neurovascular coupling with regional neurons resulting in an impaired cerebral blood flow with ischemia and hypometabolism to regional neurons. Additionally, this separation of ACfp–drAC from NVU could result in an impaired cellular crosstalk between regional neurons and their NVUs in addition to the separation of the drAC crosstalk with vascular mural cells and could result in an increased permeability. aMGC = activated microglia cell; aMt = aberrant mitochondria; Cl = capillary lumen; EC N = endothelial cell nucleus; drAC = detached astrocyte; RBC = red blood cell; Available via license CC BY 4.0.

It is important to note that others have noted ACfp detachments and separation in diabetic models in ischemia/reperfusion studies (using a fluorescence confocal scanning microscope with a 10-micrometer scale bar) and that the significant loss of ACfp/NVU may contribute to an impaired response due to a loss of communication between the NVU and regional neurons in these diabetic models [11]. Further illustrations of the ACfp detachment, separation, and retraction of the ACfp in the DBC model are provided (Figure 7).

## 5. Conclusions

The treatment with empagliflozin for 10 weeks (ages 10–20 weeks) resulted in the protection of the neuroglia ACfp from a separation, detachment, and retraction from the NVU EC and PC basement membranes. Therefore, the “6 degrees of separation” could also apply to ACfp loss of a mean of four connections (Figure 6). Importantly, the protection of the ACfp detachment, separation, and retraction we have noted at the ultrastructural level may be very novel and important. The protective effect with empagliflozin treatment appears to also possibly be related to the prevention of impaired cognition and dysfunction in these models as demonstrated by Lin B et al. [5].

A separation of ACfp would not only interfere with neurovascular coupling and impaired functional hyperemia but also allow for an increased permeability of the NVU via the loss of paracrine maintenance and regulation of the EC TJ/AJs of the BBB. Furthermore, an increased permeability of the NVU could be due to the paracrine loss/impairment of aquporin-4’s (AQP-4) effect on NVU water homeostasis, since it is known that as AQP-4 function decreases permeability increases [6,7].

A 2015 paper by Ramos-Rodriguez et al. was able to demonstrate the gross morpho-pathologic atrophy as well as a reduction in brain weights in their *db*/*db* models at 26 weeks of age as compared to the controls [12]. These models were only 6 weeks older than ours, which were sacrificed at 20 weeks of age. Therefore, we hypothesize that the ACfp structural detachment, separation, retraction, and loss of function may be a contributor to the observed loss of brain volume and neuronal loss of integrity with dysfunction and loss that would be undoubtedly associated with cognitive impairment, dysfunction, loss, and even cortical atrophy as previously mentioned [12].

T2DM in the DBC appears to have a negative modulation of information processing, in that, there develops an impaired cognition and memory [5,6]. Importantly, empagliflozin appears to have a protective modulation of information processing, in that, the impaired cognition and memory observed in the DBC models are protected, which may be due to the protection of the communicative function of the intact ACfp to the regional neurons for a modulation of information either negative as in the DBC and/or protective in the empagliflozin-treated DBE models. This experiment has only provided the specific findings and documentation that, in diabetic models, there is a detachment, separation, and retraction of the ACfp that was significant and associated with impaired cognition, which assisted in forming the hypothesis that an ACfp attachment to the outer basement membranes of the neurovascular unit may be essential for the proper modulation of information processing.

## 6. Future Directions

Hopefully, by sharing these ultrastructural findings of ACfp remodeling in the diabetic *db*/*db* DBC model, which may represent a novel diabetic astrogliopathy and the protection by empagliflozin, there can be future communication and sharing regarding the above hypothesis. It is hoped that these ultrastructural findings and hypothesis may provide for future more in-depth findings and discussions regarding the loss of the intact ACfp to the NVU. The important role of the ACfp and cerebral blood flow are to be now entertained as a possible structural and functional mechanism that may be important to the modulation of information processing, at least, in the more frontal regions of the cerebral cortex layer III in the *db*/*db* diabetic DBC brain with impaired cognition that appear to be protected with the STLT2 inhibitor empagliflozin and possibly other glucose-lowering or insulin-sensitizing medications.

## Abbreviations

AC	astrocyte
ACfp	astrocyte foot processes
BBB	blood-brain barrier
BM	basement membrane
CBF	cerebral blood flow
CKC	control: lean non-diabetic female heterozygous +/-C57B/6Jbackground
CL	capillary lumen
CNS	central nervous system
DBC	db/db homozygous +/+ obese -insulin resistant -female diabetic model
DBE	db/db models treated with empagliflozin
EC	endothelial cell
FIB/SEM	focused ion beam/scanning electron microscopy
LOAD	late onset Alzheimer’s disease
NVU	neurovascular unit
Pc	pericyte
PYR	pyramidal neuron
T2DM	type 2 diabetes mellitus
TEM	transmission electron microscopy
TJ/AJ	tight junction/adherens junction
RBC	red blood cell
SGLT2i	sodium glucose transporter 2 inhibitor
